# Electrochemotherapy with Calcium Chloride and 17β-Estradiol Modulated Viability and Apoptosis Pathway in Human Ovarian Cancer

**DOI:** 10.3390/pharmaceutics13010019

**Published:** 2020-12-24

**Authors:** Zofia Łapińska, Michał Dębiński, Anna Szewczyk, Anna Choromańska, Julita Kulbacka, Jolanta Saczko

**Affiliations:** 1 Department of Molecular and Cellular Biology, Wroclaw Medical University, 50-556 Wroclaw, Poland; anna.szewczyk@umed.wroc.pl (A.S.); anna.choromanska@umed.wroc.pl (A.C.); julita.kulbacka@umed.wroc.pl (J.K.); jolanta.saczko@umed.wroc.pl (J.S.); 2 Faculty of Pharmacy, Wroclaw Medical University, 50-556 Wroclaw, Poland; michal.debinski@student.umed.wroc.pl; 3 Department of Animal Developmental Biology, Institute of Experimental Biology, University of Wroclaw, 50-328 Wroclaw, Poland

**Keywords:** 17β-estradiol, chemotherapy, electrochemotherapy, calcium electroporation, ovarian cancer, MDAH-2774

## Abstract

Estrogens (Es) play a significant role in the carcinogenesis and progression of ovarian malignancies. Depending on the concentration, Es may have a protective or toxic effect on cells. Moreover, they can directly or indirectly affect the activity of membrane ion channels. In the presented study, we investigated in vitro the effectiveness of the ovarian cancer cells (MDAH-2774) pre-incubation with 17β-estradiol (E_2_; 10 µM) in the conventional chemotherapy (CT) and electrochemotherapy (ECT) with cisplatin or calcium chloride. We used three different protocols of electroporation including microseconds (µsEP) and nanoseconds (nsEP) range. The cytotoxic effect of the applied treatment was examined by the MTT assay. We used fluorescent staining and holotomographic imaging to observe morphological changes. The immunocytochemical staining evaluated the expression of the caspase-12. The electroporation process’s effectiveness was analyzed by a flow cytometer using the Yo-Pro™-1 dye absorption assay. We found that pre-incubation of ovarian cancer cells with 17β-estradiol may effectively enhance the chemo- and electrochemotherapy with cisplatin and calcium chloride. At the same time, estradiol reduced the effectiveness of electroporation, which may indicate that the mechanism of increasing the effectiveness of ECT by E_2_ is not related to the change of cell membrane permeability.

## 1. Introduction

Ovarian cancer (OC) is the most common gynecological malignancies associated with the highest number of deaths [1]. The International Agency for Research on Cancer (IARC) prophesies that the number of women diagnosed with OC will increase by 47% before 2040 [2]. The poor prognosis and 5-year survival rates of <30% are mainly caused by the fact that nearly 3/4 of women are diagnosed when the disease is stage III or IV FIGO (Féderation Internationale de Gynécologie et d’Obstétrique) [3]. It is attributable to several factors, including vague symptoms, not effective preventative measures, and a lack of definitive screening tools [4,5]. The disease mostly affects women between 50 and 70 years of age, and is rare in young women under 30. The first-line treatment includes primary tumor debulking surgery and six cycles of subsequent chemotherapy (CT) with cisplatin or its derivatives [4]. Around 70% of patients relapse and then do not respond to the standard treatment method [6]. The reason is, cancer cells frequently acquire secondary drug resistance as a result of repeated chemotherapy [7]. The primary drug resistance of cancer cells also continues to be a problem. Furthermore, because of the many side effects of conventional CT, there is an urgent need to search for new, more effective, and less toxic therapies.

OC belongs to estrogen-dependent malignancies because of hormones and reproductive factors’ distinct influence on the disease’s pathogenesis and progression [8]. Unfortunately, the exact molecular mechanism of this relationship remains unclear. Mungenast et al. describe that estrogens metabolism disorders may cause the formation of DNA adducts, which, together with free radicals from the metabolic activation to reactive catechol estrogens, are responsible for DNA damage [5].

The predominant intracellular estrogen is 17β-estradiol (E_2_), of which increased production is noted in many OC patients [9]. The biological activity of E_2_ is based on binding and interaction with specific receptors, namely, estrogen receptor α (ERα, also known as ER1 or Esr1), estrogen receptor β (ERβ, also known as ER2 or Esr2), and G protein-coupled estrogen receptor (GPER1, formerly known as GPR30) [10]. Most of the patients show increased expression of the ERα receptor, in contrast to ERβ, decreasing during tumor development [11]. Moreover, compounds that are antagonists of this protein exhibit a strong inhibitory effect on ovarian cells’ growth, both healthy and neoplastic cells.

The mechanism of the action of estradiol is divided into genomic and non-genomic. By a non-genomic mechanism, estrogen, through interaction with membrane ERs (mERs), may influence the activation of mitogen-activated protein kinases (MAPKs), tyrosine kinases, or G proteins, or may modulate the function of ion channels [12,13]. One of the well-known non-genomic pathways of the estradiol activity is the activation of the G protein-coupled estrogen receptor [14]. Estradiol has the highest affinity for this receptor among all estrogens. After the E_2_ binding to the receptor, the G protein is activated. That results in a signaling cascade, leading to the mobilization of calcium ions (Ca^2+^) from the endoplasmic reticulum and the generation of secondary messengers: Inositol triphosphate (IP_3_) and diacylglycerol (DAG) [12]. Activation of GPER-1 receptor could also, through the increase in the metalloproteinase production, lead to the Heparin-binding EGF-like growth factor (HB-EGF) release. HB-EGF binds to the epidermal growth factor receptor (EGFR, also known as ErbB-1 or HER1 in humans). This event results in the activation of nuclear receptors responsible for cell proliferation regulation [13,14,15]. A significant increase in the GPER-1 expression level was noted in ovarian cancer cells [12]. Unfortunately, its role has not been fully understood to date. Researchers point to increased proliferation and invasiveness of GPER-1 positive cells [14].

On the other hand, recent studies show the anti-proliferative and pro-apoptotic effect of GPER-1 stimulation, resulting from the arrest in the G2/M-phase of the cell cycle. Estrogens can also directly or indirectly influence membrane ion channels’ activity by integrating into the cell membrane [13]. This action may include increasing the frequency and length of time these channels will be open, or vice versa, completely disabling their function. Estrogens can regulate intracellular calcium ion concentration in endothelial and smooth muscle cells by inhibiting L-type calcium channels [16]. They also affect K^+^ ion concentration by opening Ca^2+^ channels and voltage-activated K^+^ channels through cGMP-dependent phosphorylation.

Estrogens (Es), depending on their concentration, can also be a useful therapeutic agent for some carcinomas. For example, its high doses were used for breast cancer treatment, while it is known that estrogens are responsible for stimulating the growth of breast cancer cells [17,18]. This situation has been named an ‘estrogen paradox’.

Electroporation (EP) is a technique in which short, high-voltage pulses induce cell membrane permeabilization by creating hydrophilic pores and reorganizing membrane lipids [19]. Depending on the electric field’s applied parameters, this process may be reversible in time or irreversible (IRE) [20,21]. Another range of pulses used in the electroporation technique are nanoseconds, where we use terms of a nanosecond pulsed electric field (nsPEF) or nanosecond electroporation (nsEP) [22]. It is based on the use of pulses with a duration below 1 μs with very high intensity (even up to 100 kV/cm). It was observed that nanosecond pulses can permeabilize and disrupt not only the outer cell membrane, but also inner membranes [23]. One of the essential features of nsEP is the fact that the death of cells electroporated in this way occurs through apoptosis [24].

The combination of electroporation with standard chemotherapy called electrochemotherapy (ECT) enables the effective delivery of low permeability chemotherapeutic agents to cells [25]. Electroporation facilitates their transmembrane transport and, as a result, increases cytotoxicity [21]. Research was carried out on many chemotherapeutic agents (i.e., paclitaxel, cisplatin, bleomycin, and carboplatin), but only two of these drugs have been identified as potential agents for use with electrochemotherapy and are currently used in clinical practice: Cisplatin and bleomycin [26,27,28,29]. The main advantage of ECT is that the doses of drugs needed to achieve a local cytotoxic effect are significantly reduced [30]. The cisplatin toxicity increases almost 80 times in this method, and in the case of bleomycin, it rises to 1000 times. ECT introduces rare side effects; it is easy to perform (the procedure takes about 30 min) and relatively cheap [20,21]. Its development is currently focused on adapting it to the treatment of broader and deeper tumors (e.g., liver, prostate) [20,31]. Unfortunately, to date, little is known about its application in ovarian cancer. In our experiments, we used settings optimized for reversible EP of tumor cells following the European Standard Operating Procedures of ECT (ESOPE) protocol [32].

Calcium electroporation (CaEP) is a new, experimental modification of electrochemotherapy, in which the chemotherapeutic agent is replaced by calcium ions (Ca^2+^) [33,34]. Calcium is a second-order messenger involved in transcription regulation, metabolism, proliferation, and cell death [35]. The concentration of free calcium in the eukaryotic cell is tightly controlled and extremely low (10^−7^ mol/L), in contrast to its concentration in the plasma (10^−3^ mol/L). Even small changes in the permeability of the membrane can significantly increase the concentration of intracellular calcium. To balance the calcium concentration, the cell shows increased Ca^2+^-ATPase activity. High intracellular Ca^2+^ concentration also causes disturbances in the permeability of mitochondrial membranes, loss of electrochemical gradient, and Na^+^/K^+^-ATPase activation. Intensive use of ATP combined with its synthesis inhibition results in a rapid depletion of its reserves and ultimately introduced the cell to the necrosis pathway. Moreover, overloading the cell with Ca^2+^ may induce the formation of reactive oxygen species (ROS) and the activation of lipases and proteases.

Apoptosis, also called programmed cell death, is a process by which a cell undergoes genetically regulated suicide death [36]. This process affects damaged or unnecessary cells, and its primary goal is to maintain homeostasis. It can be initiated by several extra- and intracellular factors. Depending on the type of factor, its amount and duration of action, as well as the type of cell, the process of apoptotic or necrotic cell death can be initiated [37]. At low concentrations, cytotoxic compounds may induce apoptosis, while at high concentrations, they will lead to necrosis. Several apoptosis pathways can be distinguished depending on the inducer, including the endoplasmic reticulum (ER) stress-induced pathway [38]. Inducing factors include disruption of ER homeostasis resulting from an accumulation of misfolded proteins, disturbances in calcium ion (Ca^2+^) balance, or the activity of reactive oxygen species (ROS). The process is associated with the caspase-12 and c-Jun N-terminal kinases (JNK1) activity. Procaspase-12, located in the ER membrane, is transformed to caspase-12 in response to stress factors. Then, caspase-3 (activated by caspase-12) initiates the execution phase of apoptosis [37,39].

The available information allows hypothesizing that electroporation may be an effective method against ovarian cancer. Additionally, the controlled concentration of 17β-estradiol may have a protective or toxic effect on the MDAH-2774 ovarian cancer cell line, which expresses estrogen receptors (ERs) [40,41]. The presented research aimed to evaluate the modulating effect of 17β-estradiol on the effectiveness of chemotherapy and electrochemotherapy with the use of cisplatin and calcium chloride in human ovarian cancer.

## 2. Materials and Methods

### 2.1. Cell Culture

The research was carried out on human ovarian endometrioid adenocarcinoma cell line MDAH-2774, purchased from ATCC^®^ (Manassas, VA, USA) originating from a patient who did not receive chemotherapy or radiation prior to the collection of cancer cells [42]. Cell culture was maintained at 37 °C, 5% CO_2_, 95% air humidity in RPMI-1640 medium (GlutaMAX^TM^ GIBCO; Thermo Fisher Scientific, Inc., Waltham, MA, USA) supplemented with 10% fetal bovine serum (FBS, Sigma-Aldrich, St. Louis, MO, USA). Cell passages were carried out 2–3 times a week when confluency was about 80–90%. Cells were then removed from the flasks by trypsinization (trypsin 0.25% and EDTA 0.02%; Sigma-Aldrich, St. Louis, MO, USA) and washed with DPBS buffer (Sigma-Aldrich, USA).

### 2.2. Examined Substances

Stock solutions of 17β-estradiol (10 mM) in 96% ethanol and calcium chloride (CaCl_2_; 100 mM) in distilled water were performed. The stock solutions were stored at 2–8 °C temperature. The stock solution of cisplatin (1 mM) in distilled water was freshly prepared each time before the experiment due to the low stability of the solution. Subsequently, the proper amount of stock was mixed with RPMI-1640 to achieve the required concentration.

### 2.3. Cell Viability Assay

The MTT assay determines the viability of the cells following treatment. It evaluates the mitochondrial activity, which functions as a marker of cell viability. Briefly, MDAH-2774 cells were seeded into 96-well microculture plates at 1 × 10^4^ cells/well and incubated with 17β-estradiol, cisplatin, and CaCl_2_ at indicated concentrations and periods. Then, 100 µL/well of the MTT [3-(4,5-Dimethylthiazol-2-yl)-2,5-Diphenyltetrazolium Bromide] reagent (Sigma-Aldrich, USA) was added, and cells were incubated 2 h at 37 °C. Formazan crystals were dissolved by adding 100 µL of acidic isopropanol (38% HCl in 99.7% isopropanol). The absorbance value was measured at 570 nm using GloMax^®^ Discover multimode microplate reader (Promega, Madison, WI, USA). The experiments were performed in three replicates. The results were expressed as the percentage of viable cells relative to untreated control cells.

### 2.4. Pulsed Electric Fields (PEF) Treatment

For electroporation, cells were trypsinized, counted, and distributed at 5 × 10^5^ cells/falcon tube (15 mL), then centrifuged (5 min, 1000 rpm, Centrifuge 5430 R, Eppendorf AG, Hamburg, Germany) and resuspended in calcium-depleted medium—SMEM (Spinner’s minimum essential medium, Sigma Merck M8167, a highly conductive medium where conductivity = 1.53 S/m) with the addition of selected concentrations of cisplatin (25 µM) and CaCl_2_ (2.5 mM). The experiment was performed in sterile polycarbonate cuvettes with a 4 mm gap between electrodes (BioRad^®^, Hercules, CA, USA) by using three pulsing protocols: (1) 1.3 kV/cm × 100 µs × 100 Hz × 8 pulses (ESOPE); (2) 37.5 kV/cm × 10 ns × 1 Hz × 200 pulses; and (3) 50 kV/cm × 10 ns × 1 Hz × 200 pulses. BTX ECM 820 (Harvard Apparatus, Holliston, MA, USA) electric pulse generator delivered microsecond pulses, and PPG-20 generator (FID Technology, Burbach, Germany) 10 ns pulses with time rise of 2 ns. After the pulse delivery, cells were incubated 10 min at 37 °C and then centrifuged (5 min, 1000 rpm). The pellet of cells was suspended in the culture medium. Then, the MTT assay, microscopic, and cytometric analysis were performed.

### 2.5. Flow Cytometry Study

The efficiency of the electroporation protocols was by assessing the amount of the Yo-Pro^TM^-1 Iodide fluorescent dye absorbed by the cell. Initially, cells were trypsinized and suspended in calcium-depleted electroporation buffer—SMEM (5 × 10^4^ cells/400 µL), and Yo-Pro™-1 Iodide (YP-1, λ_exc_491/λ_em_509, Thermo Scientific^TM^, Warszawa, Poland) was added. Cell suspensions were then transferred to cuvettes and electroporated. Then cells were washed by DPBS to remove the content of the free dye, gently centrifuged 2 min × 100× *g*, and suspended in 500 µL of DPBS for measurements in polystyrene FACS tubes, 10^4^ events/sample were measured in triplicate. Flow cytometric analysis was performed by using the CyFlow CUBE-6 flow cytometer (Sysmex, Warszawa, Poland). Data was analyzed using CyView Sofware (Sysmex, Warszawa, Polska).

### 2.6. Immunocytochemical (ICC) Staining

Immunocytochemical staining semi-quantitatively assessed the expression of caspase-12. Caspase-12 evaluates the induction of apoptosis. Cells were trypsinized and suspended in a culture medium with the addition of 10 µM 17β-estradiol so that the number of cells in suspension is equal to 5 × 10^4^ cells/mL RPMI-1640. Cultures were harvested on 12-well PTFE microscopic diagnostic slides (Thermo Scientific^TM^, Portsmouth, NH, USA) and incubated for 24 h, then exposed to cisplatin and CaCl_2_ for 24 h at 37 °C and fixed using 4% paraformaldehyde for 10 min. EXPOSE Mouse and Rabbit Specific HRP/DAB Detection IHC kit (Abcam, Cambridge, MA, USA) was used to execute immunocytochemical staining. After rinsing in PBS (Bishop) (2 × 10 min), any remnant peroxidase activity was removed by incubation with Hydrogen Peroxide Block for 10 min. Samples were permeabilized by incubation with 1% Triton X-100 (Sigma-Aldrich, USA) in PBS (Bishop). The overnight exposure to a primary antibody: Rabbit polyclonal IgG (dilution rate: 1:100; SC-5627; Santa Cruz Biotechnology, Santa Cruz, CA, USA) at 4 °C visualized the expression of caspase-12. Then the secondary antibody conjugated with horseradish peroxidase (HRP) was added to samples. The next step was incubation with diaminobenzidine–H_2_O_2_ mixture to visualize the HRP label and hematoxylin (Alchem, Toruń, Poland) for 1 min to stain cell nuclei. Slides were dehydrated using ethanol (Chempur, Piekary Śląskie, Poland) gradient (6 × 5 min) and xylene (Chempur, Poland) (3 × 5 min) and covered using DPX gel (Aqua-Med Zpam-Kolasa, Łódź, Poland). The upright microscope (Olympus BCX43, Warszawa, Poland) was used to examine the immunocytochemical reaction. The intensity of immunohistochemical staining was evaluated as (−) negative, (+) weak, (++) moderate, or (+++) strong.

### 2.7. Confocal Laser Scanning Microscopy (CLSM) Study

Cytoskeleton confocal microscope evaluated modifications in the structure of MDAH-2774 cells. Cells were suspended (5 × 10^4^ cells/mL) in a culture medium, seeded into coverslips placed on a 6-well plate, and incubated for 24 h, 37 °C. Next, selected concentrations of cisplatin and CaCl_2_ were added and incubated for 24 h, 37 °C. Then, cells were fixed in 4% paraformaldehyde for 10 min, raised in PBS (3 × 5 min), blocked with 1% Bovine Serum Albumin (BSA; Sigma-Aldrich, St. Louis, MO, USA) in PBS for 1 h, and permeabilized using 1% Triton X-100 in PBS (3 × 5 min). After 1 h incubation with a solution of the Alexa Fluor^TM^ 546 phalloidin (Life Technologies-Thermo Fisher Scientifi, Waltham, MA, USA) in PBS (dilution rate: 1:300), cells were raised in PBS (3 × 5 min), then were mounted in a fluorescence mounting medium (Fluoromount^TM^ Aqueous Mounting Medium, Sigma-Aldrich, USA). The assessment of changes in the cytoskeleton structure was made based on images taken with Olympus FluoView FV1000 confocal laser scanning microscope (Olympus, Tokyo, Japan).

### 2.8. Living Cell Tomographic Microscopy

The experiment aimed to evaluate the morphological changes in MDAH-2774 cells after 24 h incubation with cisplatin and CaCl_2_, depending on whether or not cells have been incubated with 10 µM 17β-estradiol. Firstly, cells were trypsinized and suspended in a culture medium (5 × 10^4^ cells/mL). Suspensions of cells were then seeded into ibidi µ-Dish^35mm^ Quad imaging dishes and incubated 24 h, 37 °C. The next day, cisplatin and CaCl_2_ were added, and the 24 h incubation was repeated. Visualization of the 3D live cell morphology of MDAH-2774 cells was performed using a live cell tomographic holographic 3D microscope Nanolive (3D Cell Explorer, Lausanne, Switzerland).

### 2.9. Statistical Analysis

The experiments were performed in 3 replicates. The statistical analysis was performed using the GraphPad Prism 8 (GraphPad Software Inc, San Diego, CA, USA). Data are expressed as mean ± SD (standard deviation) of the mean and were analyzed by two-way ANOVA (analysis of variance), with *p* < 0.05 being considered statistically significant.

## 3. Results

### 3.1. Cytotoxicity of the Examined Substances and IC_50_ Determination

**Figure 1:**
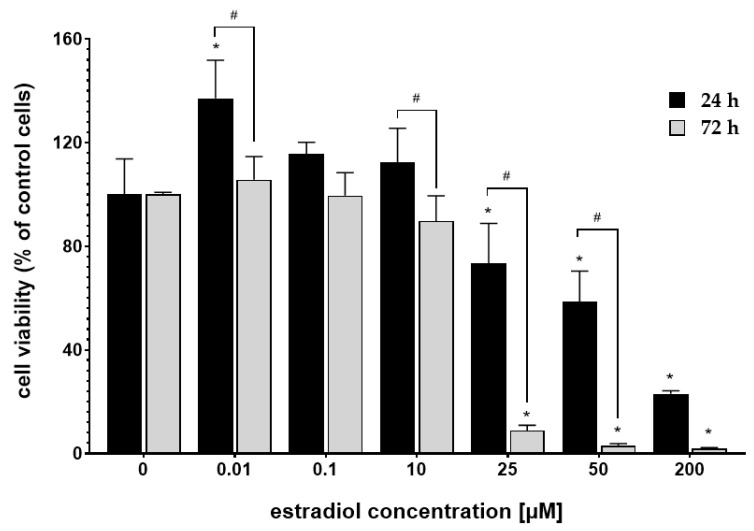
MDAH-2774 cells viability measured by the MTT assay after 24 h and 72 h incubation in different 17β-estradiol concentrations. Notes: (mean ± SD) *N* = 3, * *p* < 0.05 compared to control, # *p* < 0.05 compared between different incubation times.

MTT assay assessed the cytotoxicity of the 17β-estradiol (E_2_), cisplatin, and calcium chloride (CaCl_2_). Figure 1 shows the response of MDAH-2774 cells to the 24 h and 72 h incubation with E_2_. After 24 h incubation, E_2_ intensified cytotoxic activity in concentration 25–200 µM. However, extended incubation time (72 h) further decreased cells’ viability. Estradiol in concentrations 0.01–0.1 µM did not significantly change the cells’ mitochondrial activity compared to the untreated control. Based on the results presented above, the low-toxic concentration of 10 µM was selected for further experiments.

**Figure 2:**
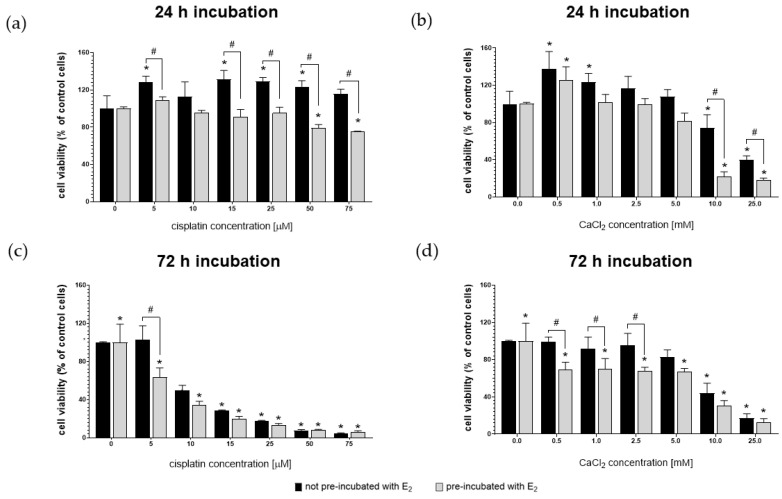
MDAH-2774 cells viability measured by the MTT assay after 24 h (**a**,**b**) and 72 h (**c**,**d**) incubation in increasing cisplatin (**a**,**c**) and CaCl_2_ (**b**,**d**) concentrations. Notes: (mean ± SD) *N* = 3, * *p* < 0.05 compared to control, # *p* < 0.05 compared between different pre-incubation conditions.

Figure 2 shows the viability of MDAH-2774 cells after 24 h and 72 h exposure to increasing concentrations of cisplatin and CaCl_2_. The experiment was carried out on two groups of cells. One of them was prior pre-incubated for 24 h with 10 µM 17β-estradiol (E_2_). Control represents the viability of untreated cells.

After 24 h exposure, MDAH-2774 cells were resistant to the cytotoxic effects of cisplatin in the full range of concentrations (Figure 2a). Cells pre-incubated with 17β-estradiol had an increased sensitivity to both compounds. However, the decrease in the mitochondrial activity of pre-incubated cells at the two highest levels of cisplatin (50 µM and 75 µM) administered was a maximum of ~26%. In contrast, the viability of pre-incubated cells after the exposure to 10 mM and 25 mM of CaCl_2_ was ≤20%. Extending the incubation time resulted in a more substantial cytotoxic effect of cisplatin (Figure 2b). In concentrations of 10–25 µM, it had a stronger than CaCl_2_ cytotoxic effect on both groups of cells. Table 1 presents the IC_50_ values calculated for cisplatin and CaCl_2_. The IC_50_ values calculated using Quest Graph™ IC_50_ Calculator (AAT Bioquest, Inc, Sunnyvale, CA, USA). IC_50_ was determined with a non-linear model.

Based on the obtained results, the highest non-toxic concentrations of cisplatin and CaCl_2_ were selected for subsequent experiments, amounting to 25 µM and 2.5 mM, respectively.

### 3.2. Effect of Pulsed Electric Fields (PEF) on Cancer Viability

**Figure 3:**
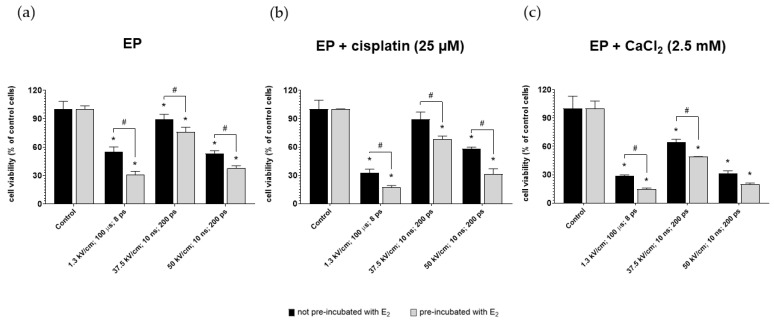
MDAH-2774 cells viability measured by the MTT assay after (**a**) µEP and nsEP with (**b**) 25 µM cisplatin and (**c**) 2.5 mM CaCl_2_. Notes: (mean ± SD) *N* = 3, * *p* < 0.05 compared to control, # *p* < 0.05 compared between different pre-incubation conditions; ps—pulses.

The viability of ovarian cancer cells after µEP and nsEP was evaluated for 25 µM cisplatin and 2.5 mM calcium concentrations. The experiment was performed on the two groups of cells (pre-incubated and not pre-incubated with 10 µM of 17β-estradiol). Figure 3 shows the viability of MDAH-2774 cells after exposure to µEP and nsEP with calcium ions or cisplatin relative to control. Control included non-electroporated cells. For both compounds, pre-incubation with E_2_ improved the cytotoxic effect of electroporation in all electric field strengths. The highest values of mitochondrial activity for both compounds occurred by electroporated cells according to the nanosecond protocol with an electric field strength of 37.5 kV/cm. The lowest viability was achieved for the microsecond electroporation protocol. The use of 2.5 mM calcium ions instead of a cytostatic gave a more significant cytotoxic effect (Figure 3c).

### 3.3. Caspase-12 Expression

**Figure 4:**
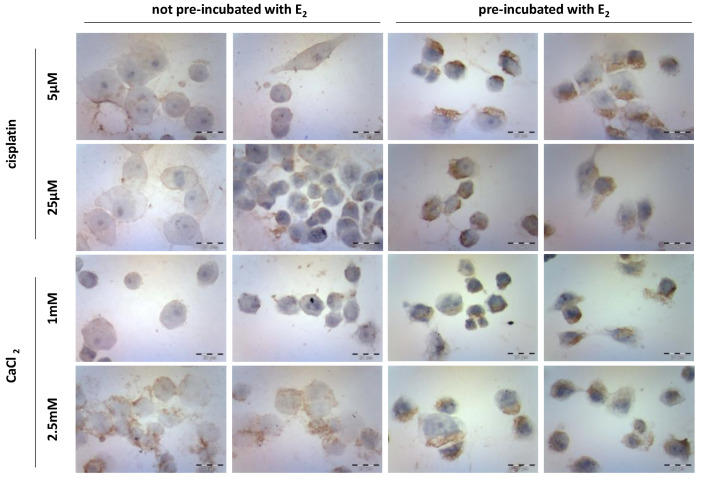
Caspase-12 immunocytochemical staining after exposure to cisplatin (5 µM and 25 µM) and CaCl_2_ (1 mM and 2.5 mM) in the MDAH-2774 cell line.

Immunocytochemical staining studies (Figure 4) revealed the expression of caspase-12 in MDAH-2774 cells after 24 h incubation with cisplatin (5 µM and 25 µM) and CaCl_2_ (1 mM and 2.5 mM). The study was performed on two groups of cells, not pre-incubated and pre-incubated with 10 µM of 17β-estradiol (E_2_) for 24 h. Controls included untreated cells. The majority of not pre-incubated cells exposed to both cisplatin concentrations remained almost unaffected. The highest level of enzyme expression was observed in cells incubated with 2.5 mM CaCl_2_. Pre-incubation of cells with E_2_ increased the expression of caspase-12 by an average of 10% in all variants of the used compounds. Cells exposed to calcium chloride showed the highest level of expression. Its significant increase was noted for the CaCl_2_ concentration equal to 1 mM. Table 2 presents the % ratio of stained cells to the whole number of cells in the sample.

### 3.4. Fluorescence Staining of Actin

**Figure 5:**
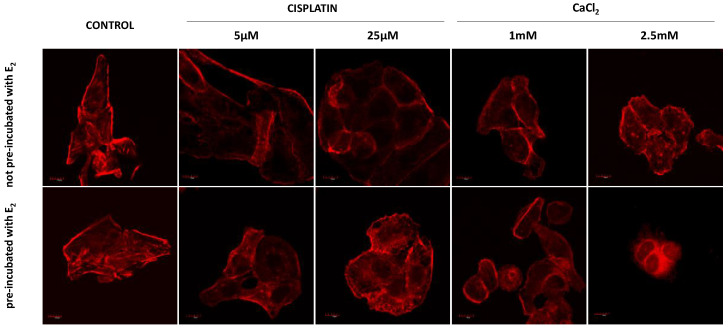
Immunofluorescence staining of MDAH-2774 cells (60×) after 24 h incubation with cisplatin and CaCl_2_. Alexa Fluor^TM^ 546 phalloidin used for actin filaments labeled. Scale bars correspond to 100 µm.

**Figure 6:**
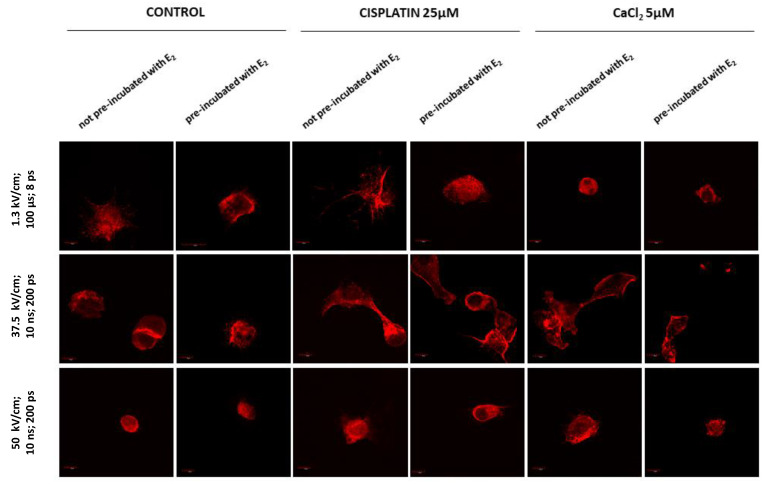
Immunofluorescence staining of MDAH-2774 cells (60×) after µEP and nsEP with the addition of cisplatin and CaCl_2_. Alexa Fluor^TM^ 546 phalloidin used for actin filaments labeled. Scale bars represent 100 µm; ps—pulses.

Figure 5 and Figure 6 present fluorescence staining of the intracellular actin in MDAH-2774 cells. The studies were obtained for two groups of cells, not pre-incubated and pre-incubated with 10 µM of 17β-estradiol (E_2_) for 24 h. Cells were electroporated using µEP and nsEP protocols (Figure 6). Controls included non-electroporated and untreated samples.

Control cells formed elongated-shape, well-organized F-actin filaments structure, creating a stable net with stress fibers. Both concentrations of cisplatin did not result in significant changes in the morphology of the neoplastic cells (Figure 5). The most significant changes are seen in cells pre-incubated with E_2_ and then exposed to 25 µM cisplatin. The cells changed shape to oval. The actin filaments accumulated near the nucleus and on the edge of the cell in lamellipodia and filopodia. Exposure to calcium chloride-induced more substantial changes in cell morphology and the cytoskeleton. Significant shrinkage appears at a concentration of 1 mM, while at 2.5 mM CaCl_2_, cells have an abnormal structure and probable damage to the actin cytoskeleton (pre-incubated cells).

Electroporation without the addition of any compound caused severe morphological changes (Figure 6). They are manifested in most attempts by significant cell shrinkage. The most potent effect was observed after microsecond (µEP) and nanosecond (nsEP) electroporation in particular for an electric field strength of 50 kV/cm, where cells revealed inferior assembly of actin filaments. Reorganization of actin cytoskeleton to both pre-incubated and not pre-incubated cells is visible in all these sample. Nanosecond electroporation with the intensity of 37.5 kV/cm resulted in changes in the cytoskeleton structure such as extending lamellipodia-like protrusions, which provided cell–cell contact and migration ability. However, no cell shrinkage was noted. The differences between not pre-incubated and pre-incubated cells are particularly visible in non-electroporated samples. Cells pre-incubated with E_2_ are smaller, more oval, or damaged (2.5 mM CaCl_2_).

### 3.5. Efficiency of Microsecond Electroporation (µEP) and Nanosecond Electroporation (nsEP)

**Figure 7:**
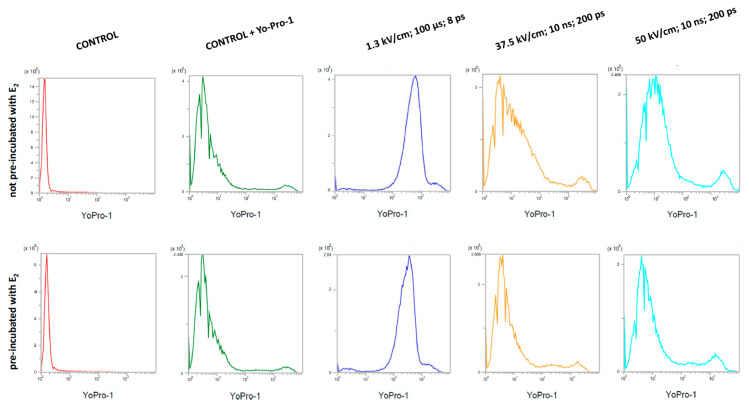
Yo-Pro-1^TM^ fluorescent dye absorption in MDAH-2774 cells after µEP and nsEP; ps—pulses.

The uptake of the Yo-Pro-1^TM^ fluorescent dye shows the efficiency of the electroporation process (Figure 7). The dye fluorescence intensities were measured with a flow cytometer. The study included two groups of cells (not pre-incubated and pre-incubated with 10 µM 17β-estradiol for 24 h). Non-electroporated controls with and without the addition of the dye were included.

The highest absorption values of the Yo-Pro-1^TM^ dye were found in cells subjected to microsecond electroporation with an electric field strength of 1.3 kV/cm. The efficiency of nsEP was much lower. At an electric field strength of 37.5 kV/cm, the mean value of fluorescence was similar to the non-electroporated control. After electroporation with the nsEP 50 kV/cm protocol, the dye absorption reached intermediate values. Pre-incubation of cells with 17β-estradiol reduced the uptake of the dye in all the tests performed. Table 3 shows the mean values of fluorescence intensity obtained during the study.

### 3.6. Digital Holographic Microscopy (DGM) Study

**Figure 8:**
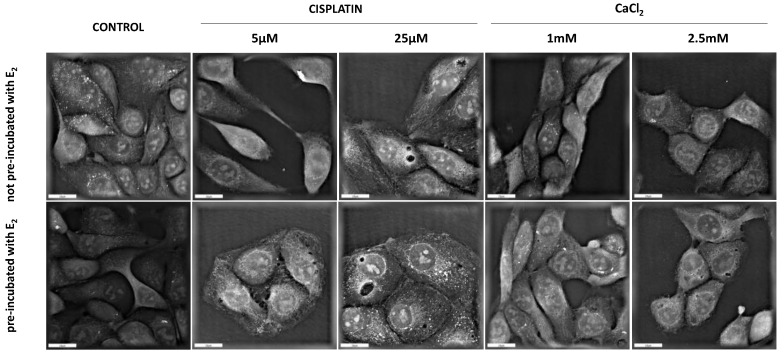
Holotomographic microscopy studies after 24 h with cisplatin and CaCl_2_. Scale bars represent 20 µM.

Digital Holographic Microscopy (DGM) study showed the morphological changes in the MDAH-2774 cell line after 24 h incubation with examined compounds. The experiment included two groups of cells (not pre-incubated and pre-incubated with 10 µM of 17β-estradiol for 24 h). Controls incorporated untreated samples. Figure 8 presents the obtained results. The control cells are in good condition and have a normal structure. There are no significant differences between pre-incubated and not pre-incubated controls. The most remarkable changes in cell structure are noticeable after exposure to calcium chloride at a concentration of 2.5 mM. In this case, both series of cells are visibly shrunken and take a more oval shape. Incubation with cisplatin did not provoke significant morphological changes. However, a difference can be observed between the series of cells exposed to this compound.

## 4. Discussion

Neoplastic diseases are currently one of the most common causes of death in the world. Ovarian cancer (OC) is associated with the highest death rate out of any gynecological malignancies. The disease in the early stages of development gives no or vague symptoms. It is estimated that ~75% of women are diagnosed in stage III or IV FIGO. Apart from late diagnosis, treatment failure is caused by frequent relapses and the phenomenon of patient resistance to the drugs used. High hopes in the fight against neoplasms resistant to the currently used therapeutic strategies are given by electrochemotherapy (ECT). This method allows for an extreme increase in the absorption of cytostatics by cancer cells and, as a result, intensification of their action. ECT has already been used successfully in patients who do not respond to conventional chemotherapy [43,44].

Moreover, it is characterized by a safety profile and a lower frequency of oppressive contingent effects because of its local activity. Hitherto, ECT has been approved in the treatment of skin cancers and head and neck cancers. Additionally, it is expected that it may find application in treating deep cancers in the future. Even greater expectations are associated with calcium electroporation (CaEP), where calcium ions (Ca^2+^) replace conventional cytostatic. Considering the low efficacy of current treatments for OC, studies on the effectiveness of different types of electrochemotherapy and their impact on the biology of ovarian cancer cells are fully justified.

The molecular mechanisms associated with ovarian cancer are closely related to the action of estrogen hormones. Their direct action on ovarian cells may affect tumor development and its progression. This is reflected in the wide range of risk factors associated with estrogens’ effects (e.g., hormone replacement therapy). Estrogens, by enhancing proliferation, can cause the consolidation and accumulation of DNA damage. Apart from participation in neoplastic transformation, estrogens may have a protective effect on ovarian malignancy cells, inhibiting the apoptosis process. However, according to the studies conducted so far, estrogens’ impact may differ depending on their concentration, the type of target cells, and the expression of particular estrogen receptor (ER) types. Considering the potential use of electrochemotherapy in the treatment of ovarian cancer, the above study posed the question of whether 17β-estradiol (E_2_; the most active of estrogens) has a protective effect on electroporated ovarian cancer cells or, on the contrary, will intensify the toxic effects of cisplatin and calcium chloride (CaCl_2_).

The first stage of our research was to estimate the cytotoxicity of equal concentrations of estradiol and its influence on the compounds’ effectiveness in the subsequent phases during ECT. We analyzed cisplatin, a drug commonly used in OC therapy, and CaCl_2_, a calcium ion source for the experimental calcium electrochemotherapy. The cytotoxicity profile of the mentioned compounds was determined for 24 and 72 h of incubation.

Estradiol reduced the mitochondrial activity of MDAH-2774 cells in the concentration range of 25–200 µM. These are concentrations exceeding the physiological values. This effect was particularly visible after 72 h of incubation. No cytotoxic effect was observed at concentrations of 0.01–10 µM. Zhenga et al. pointed out that 6-day incubation of OVCAR-3 cells with 0.01–100 nM E_2_ intensifies their proliferation [45]. Additionally, Langdon et al. showed analogous tendencies in PEO1 and PEO4 cell lines, and it was associated with the Erα receptor activity [46]. On the other hand, Bonavida and Bechtel demonstrated the anti-proliferative effect of estradiol at concentrations of >1 µM in HOC-7 and OVCAR-3 cells [47]. Based on the performed MTT assay, the concentration of 17β-estradiol used for the pre-incubation of cells in subsequent experiments (10 µM) was selected. This is the concentration that was not cytotoxic after 24 h of expansion and is the closest to the physiological E_2_ concentration in the follicles of the ovaries [48].

To assess the influence of estradiol on the studied phenomena, all experiments were performed on two groups of cells, namely non- and pre-incubated, for 24 h with the selected concentration of E_2_. Assessment of cisplatin’s effect on MDAH-2774 cells was performed using a concentration range of 5–75 µM. After 24 h of incubation, cells showed no decreased mitochondrial activity over the entire range of drug concentrations. The prolongation of the exposure time caused that the cytotoxic effect was noticeable at concentrations > 5 µM. In turn, calcium chloride’s action was assessed for concentrations in the range of 0.5–25 mM. 24 h incubation with CaCl_2_ < 5 mM did not reduce cell viability. At concentrations of 10 and 25 mM, a distinct cytotoxic effect was noted. After 72 h of exposure, 5 mM cisplatin was also toxic. Pre-incubation with E_2_ increased the susceptibility of cells to the action of both compounds.

Fluorescent staining of the actin cytoskeleton visualized the effects of cisplatin and CaCl_2_ (depending on the cells’ prior exposure to estradiol). It allowed evaluating changes in cell morphology caused by the action of the analyzed compounds probably. Two cisplatin concentrations (5 and 25 µM) and calcium chloride (1 and 2.5 mM) were selected for the study. Concentrations that were not toxic during the 24 h incubation were used to fix the cells to the microscope slides. Cells exposed to 5 and 25 µM of cisplatin showed slight changes in cell morphology and cytoskeleton. However, they were most prominent in cells pre-incubated and then exposed to cisplatin at a concentration of 25 µM. In turn, the action of CaCl_2_ caused a marked shrinking of cells. Pre-incubated cells enhanced this effect.

The mechanism of 17β-estradiol action on OC cells is mostly related to the estrogen receptors (ERs). ERα and Erβ act differently, and the level of their expression depends on the type of cells. Both types are present in the ovaries’ normal and cancer cells, but their quantitative ratio is changed during carcinogenesis. Chan et al. showed that the majority of patients with OC show increased expression of ERα and ERβ5 nuclear receptors and decreased expression of ERβ1 and cytoplasmic ERα receptors [49]. The activity of the ERα is associated with the phenomenon of cisplatin resistance, as shown in the studies conducted by Matrumur et al. [50].

Moreover, the ERβ may exert anti-tumor activity in response to or independently of E_2_ stimulation, as reported by Treeck et al. [51]. The action of sex hormones may influence the number of ERs. Taube et al. showed that estradiol in nanomolar concentrations (nM) could lower the total amount of estrogen receptors presented by ovarian cancer cells [52]. The synergistic effect of E_2_ and the compounds tested by us may be associated with a disturbance in the quantitative ratio of individual types of ERs. To confirm this hypothesis, additional experiments are required.

Another mechanism that may participate in estradiol’s observed effect may be the mechanism related to the excessive production of reactive oxygen species (ROS). Moghadasi et al. have shown that estradiol in concentrations of 0.1–1000 nM increases ROS synthesis in OVCAR-3 human ovarian carcinoma cells [53].

Caspase-12 expression was assessed to determine if decreased survival of MDAH-2774 cells pre-incubated with estradiol is associated with increased ROS synthesis. Caspase-12 is involved in the process of reticular apoptosis and is activated in response to stress factors such as, for example, reactive oxygen species or high levels of calcium ions. Immunocytochemical staining showed increased caspase-12 expression in cells pre-incubated with estradiol. However, this effect differed depending on the compound used. In cisplatin (5 and 25 µM), the number of stained cells increased by ~10%. For slides with 1 mM CaCl_2_, this value increased by 45%. Cells treated with 2.5 mM CaCl_2_ were ~95% stained regardless of pre-incubation with estradiol. Sapkal showed that 20 and 40 nM of E_2_ decreased the expression of caspase-12 in human retinal epithelial cells (ARPE-19) [54]. This effect has been linked to the antioxidant activity of 17β-estradiol. Similar results were obtained by Guo et al., studying the impact of nanomolar estradiol concentrations on the course of stress-induced apoptosis in MC3T3-E1 mouse osteoblast cells [55]. They showed that E_2_ at these concentrations inhibited reticular apoptosis by increasing the expression of Grp78, which is a protein capable of binding procaspase-7 and procaspase-12. Unfortunately, there are no reports in the available literature concerning the effect of micromolar (µM) concentrations of estradiol on ovarian cancer cell lines.

The next stage of the research was to assess the effect of 17β-estradiol on the electroporation (EP) process of MDAH-2774 cells. The study was divided into two parts. In the first one, changes in the mitochondrial activity and the structure of the cytoskeleton were examined. The second part assessed the effectiveness of electroporation by checking the absorption of the Yo-Pro™-1 fluorescent dye.

Three electroporation protocols were used, plus cisplatin (25 µM) and CaCl_2_ (2.5 mM). Calcium chloride induced a more significant decrease in cell viability than cisplatin, regardless of the used protocol. Microsecond electroporation (µEP) without any compound achieved a robust cytotoxic effect (reducing cell viability by ~50%). The addition of cisplatin and CaCl_2_ intensified this effect. The use of nanosecond pulsed electric fields (nsEP) 37.5 kV/cm did not cause a significant decrease in survival in control without compounds and in combination with cisplatin.

In contrast, the combination with CaCl_2_ caused a reduction in mitochondrial activity by ~30%. The nsEP 50 kV/cm protocol in control and combination with cisplatin caused a comparable decrease in the cell viability to a value ~55%. When combined with calcium chloride, the toxic effect was similar to that obtained with the µEP.

All electroporated cells pre-incubated previously with 17β-estradiol showed reduced mitochondrial activity. Fluorescent staining of actin filaments displayed morphological changes in cells electroporated using µEP and nsEP 50 kV/cm. The cells showed shrinkage and damage to the cytoskeleton. The nsEP 37.5 kV/cm did not cause such intense changes. The results obtained in this study for cells not pre-incubated with estrogen hormone are consistent with the literature data. Saczko et al. demonstrated that the combination of cisplatin with µEP significantly increases the toxicity of cytostatics on ovarian cancer cells [56].

Chenguo et al. presented that the mobilization of intracellular Ca^2+^ causes cytotoxicity to OC cells after nsEP [57]. The high concentration of CaCl_2_ activates apoptosis. In our study, nsEP, in combination with CaCl_2,_ caused the most substantial decrease in cell viability. Unfortunately, there are currently no publications available on the effects of E_2_ on EP. For this reason, the last of the experiments carried out in this study was to determine the impact of pre-incubation with estradiol on the efficiency of electroporation.

MDAH-2774 cells were also electroporated with Yo-Pro™-1. The absorption was measured using a flow cytometer. The study showed that µEP had the most substantial dye uptake. Cells pre-incubated with estradiol showed a lower dye absorption. Considering the results of MTT assay, it can be assumed that the mechanism behind the decrease in the mitochondrial activity of cells pre-incubated with E_2_ is not related to changes in the permeability of the cell membrane.

## 5. Conclusions

The results presented in this work demonstrated that electrochemotherapy (ECT) might be a potentially effective treatment for human ovarian cancer in vitro. Pre-incubation of ovarian cancer cells with 17β-estradiol effectively enhanced the chemo- and electrochemotherapy with cisplatin and calcium chloride. Therefore, their impact should be considered when developing new schedules of treatment.

## Figures and Tables

**Table 1:** IC_50_ values for cisplatin and calcium chloride (CaCl_2_).

	Cisplatin [µM]	CaCl_2_ [mM]
IC_50_ for 24 h		
Not pre-incubated with E_2_	727.47	20.03
Pre-incubated with E_2_	129.53	13.29
IC_50_ for 72 h		
Not pre-incubated with E_2_	9.567	16.637
Pre-incubated with E_2_	6.072	8.503

**Table 2:** The caspase-12 expression after exposure to cisplatin (5 µM and 25 µM) and CaCl_2_ (1 mM and 2.5 mM) in the MDAH-2774 cell line.

	Not Pre-Incubated with E_2_		Pre-Incubated with E_2_	
	Percentage of Stained Cells	The Intensity of Staining	Percentage of Stained Cells	The Intensity of Staining
cisplatin				
5 µM	74%	+	81.5%	++
25 µM	80%	+	90%	++
CaCl_2_				
1 mM	44.5%	+/−	90.5%	++
1.5 mM	95%	++	92%	++/+++

(−) negative, (+) weak, (++) moderate, or (+++) strong.

**Table 3:** Yo-Pro-1^TM^ fluorescence intensity in MDAH-2774 cells in arbitrary units; ps—pulses.

	Not Pre-Incubated with E_2_	Pre-Incubated with E_2_
Control	1.52	1.56
Control + Yo-Pro-1^TM^	5.32	4.97
EP 1.3 kV/cm (ESOPE)	677.19	403.98
nsEP 37.5 kV/cm (10 ns, 200 ps)	23.07	6.24
nsEP 50 kV/cm (10 ns, 200 ps)	256.7	171.46

## Data Availability

The data presented in this study are available on request from the corresponding author. The data are not publicly available due to the consent provided by participants on the use of confidential data.
